# Immersive virtual reality rehabilitation after lower limb surgery in paediatric patients

**DOI:** 10.1177/18758894241313093

**Published:** 2025-01-31

**Authors:** Ivan Phelan, Alicia Carrion-Plaza, Penny Jayne Furness, Jack Parker, Nicolas Nicolaou, Paul Dimitri

**Affiliations:** 1 Sheffield Creative Industries Institute, College of Social Sciences & Arts, Sheffield Hallam University, Sheffield, UK; 2 Department of Psychology, Sociology and Politics, College of Social Sciences and Arts, Sheffield Hallam University, Sheffield, UK; 3 Human Sciences Research Centre, College of Science and Engineering, University of Derby, Derby, UK; 4 Sheffield Children's NHS Foundation Trust, Sheffield Children's, Sheffield, UK

**Keywords:** immersive virtual reality, physical rehabilitation, lower limb surgery, paediatric, pain, anxiety

## Abstract

**Purpose:**

Patients who have undergone lower limb surgery require rehabilitation to regain movement and function in the affected leg. Unfortunately, physical rehabilitation can be painful, reducing compliance and recovery. This feasibility study aimed to demonstrate that immersive virtual reality (IVR) applications can provide potential benefits of anxiety reduction and pain distraction for children during gait rehabilitation, increased engagement and enjoyment, and improved perceived walking quality.

**Methods:**

This study included 15 children aged 11–16 who required weight-bearing rehabilitation following lower limb surgery. A mixed methods (quantitative and qualitative) approach and a multidirectional perspective (patients, parents and physiotherapists) were adopted to measure. Changes in anxiety (General Anxiety Disorder-7) and pain (visual analogue scale) before and after the intervention were assessed. Qualitative data were collected through interviews with children, their parents, and physiotherapists, focusing on their experiences, satisfaction, perceived effectiveness, and acceptability of the IVR intervention.

**Results:**

Results demonstrated that IVR for rehabilitation after lower limb surgery in children (1) reduced anticipatory anxiety; (2) reduced the level of pain experienced during gait rehabilitation; (3) improved rehabilitation, such that children were walking more than expected and with better quality; (4) increased confidence; (5) made rehabilitation more enjoyable; and (6) was delivered via a system that was easy to learn and accept.

**Conclusion:**

This rehabilitation IVR is the first product of its class for paediatric lower limb postoperative rehabilitation. These preliminary results will inform improvements to the system in a future multi-site study with a large calculated sample size to demonstrate its clinical effectiveness and safety in acquiring medical device markings and adoption.

## Introduction

Lower limb conditions requiring post-surgical rehabilitation in children include trauma and motor disabilities. National Health Service (NHS) hospitals in England registered 23,197 paediatric orthopaedic admissions in 2021–2022, with 16,219 for lower limb treatment. This was an increase on the previous year's 11,183, reflecting the impact of COVID-19.^1^ The aetiology of lower limb paediatric orthopaedic conditions varies (congenital, neuromuscular and skeletal dysplasias), but the associated motor disabilities are often similar (deformity, joint stiffness, pain and impaired mobility).^2–5^

Lower limb surgery and rehabilitation to correct function are associated with intense pain, which reduces rehabilitation compliance and recovery.^6–10^ Early mobilisation helps to accelerate rehabilitation and avoid long-term conditions such as chronic pain.^11–13^ However, children often fear walking due to anticipated and actual pain.^14^

Immersive virtual reality (IVR) has been increasingly used in patients with burns or cerebral palsy, to promote distraction from painful procedures and reduce anxiety during upper limb rehabilitation.^15–23^ IVR helps children carry out painful and repetitive rehabilitation exercises by shifting their attention away from incoming painful sensory signals and maximising recovery through increased motivation and adherence.^24,25^ IVR offers computer-generated three-dimensional (3D) scenarios where children can physically interact using a head-mounted display (HMD) and controllers. Used at home or in the clinic, IVR allows them to practice movements in a safe, engaging and challenging environment.^15^

Previous studies have utilised IVR to support targeted rehabilitation to improve lower limb function in children, but evidence of its efficacy is limited.^26–28^ Most studies focus on adults and non-immersive screen-based VR.^29^ Adult studies support IVR feasibility for balance or gait training,^26–28,30–34^ and one study involving children with neuromotor disorders demonstrated similar gains through IVR.^35^

Based on a literature review, no studies have implemented a co-designed IVR rehabilitation system tailored to the needs of children after lower limb surgery.

The system presented in this study offered an immersive rehabilitation experience for children, focusing on lower limb mobilisation and walking recovery while distracting them from pain. Children's physical features were taken into account as anatomical measures to design the mechanics of the game, matching the required rehabilitation exercises recommended by the clinicians. In addition, the narrative and scenarios were tested with the children to present visuals that appealed to their age group.

This feasibility study aimed to explore the perceptions and impacts of using a bespoke IVR game for lower limb rehabilitation for paediatric patients in a clinical setting. It investigated the potential experiential benefits such as anxiety reduction, pain distraction, and increased engagement and enjoyment, as well as the potential effectiveness of the intervention on gait rehabilitation in a clinical setting.

## Method

### Ethical considerations

Ethical approvals were obtained from HRA and Health and Care Research Wales (HCRW) (IRAS Ref. 291012) and Sheffield Hallam University (SHU) Ethics Review Committee (Ref. ER29677226). As a single site study, the site-specific approval was received from SCH NHS Hospital Trust Research and Development (R&D) (Ref. SCH-2396). All patients and parents gave written informed consent before participation, in accordance with the ethics guidelines and principles of the Declaration of Helsinki.

### Participants

The clinicians at SCH enrolled 15 participants, as this was the estimated recruitment rate that was considered to be achievable within the study time. Children aged 11–16 who required weight-bearing rehabilitation following lower limb surgery were recruited and consented. The exclusion criteria included being unsafe to weight-bear, face or head injuries that risked pain or infection when using the headset, learning impairments that could hinder the understanding of the task, or a history of severe motion sickness/vertigo.

Parents of the children and the two physiotherapists (PTs) participating in the study also consented to share their perceptions of the IVR rehabilitation system.

### Intervention and procedures

Co-design was carried out to better understand the needs of the patients and clinical staff, ensuring that the IVR system was fit for rehabilitation. These design decisions were implemented iteratively until the system was complete. A clinical trial was conducted with a final version of the system.

#### IVR system

The co-design work involved two researchers, three game developers, a consultant orthopaedic surgeon, a physiotherapist, and a Patient and Public Involvement representative (PPI). A workshop was initially conducted with two researchers and a PPI who had experienced lower limb surgery and the rehabilitation pathway in his teens, 5–7 years previously. The PPI provided detailed insight into his experience of surgery and rehabilitation and contributed to the design concepts. Observations of patients undergoing lower limb rehabilitation took place at the Sheffield Children's NHS Foundation Trust (SCH) with IP and the physiotherapy team. This provided insight into the movements involved during rehabilitation to inform system design, which included recommendations such as the required duration of the rehabilitation and avoiding excessive fatigue. As a result, the scenario was designed to be exciting, with multiple layers of feedback to maintain the patient's focus. High levels of visual and audio feedback were provided with a narrative that enhanced the immersion. These provided a focus on the virtual tasks to encourage the user to continue walking forward.^36,37^ The PPI participant and the observed group were not involved in the main study.

Equipment included an Oculus Quest 2 HMD with touch controllers and 3D printed holsters for the controllers. Potential contraindications (e.g., dizziness and seizure disorders, prolonged exposure time) were taken into account in both the inclusion criteria and the protocol. The software was comprised of Unreal Engine 4.24, 3ds Max 2021 and Substance 3D Designer 11.3.

**Figure 1.**
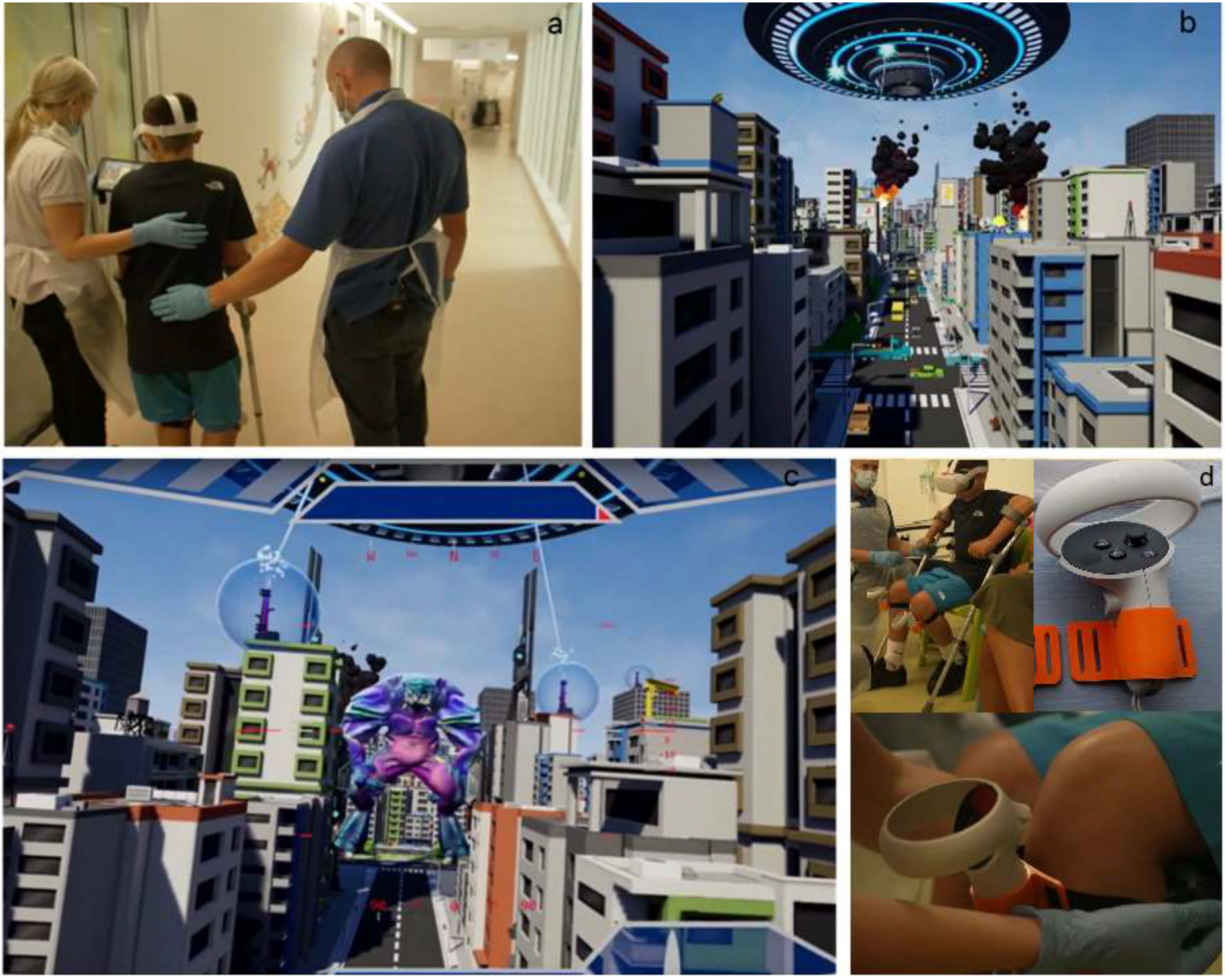
Immersive virtual reality (IVR) system. a The physiotherapists walked with patients to the end of a hallway for each session (approximately 20 metres). b Within the VR, the corridor was represented as a city street. c The scenario came to an end by combatting a giant monster with a small puzzle. d The patient was fitted with 3D printed holders with straps to attach to their legs so they could be tracked by the headset.

The system required the patient to be fitted with 3D printed holders with straps to attach the controllers to their legs so that their movement could be tracked by the headset (Figure 1d). A calibration process was undertaken with each patient while lying down, with the VR system adjusted to account for differences in leg length. Once in IVR, they were transported to a room where they could choose designs for their avatar and be informed about the mission details. Patients were then presented with a city environment in which they could control the movement of a giant robot by walking.

In keeping with the goals of rehabilitation, the physiotherapist needed patients to walk to the end of the hospital hallway in each session (approximately 20 metres). Within the VR, the corridor was represented as a city street. When the patient physically walked down the corridor, they also walked down the virtual city street (Figure 1a and b). The potential for recording the distance walked was considered at the design stage. However, the leg sensors would not be sufficiently accurate to provide reliable output data. Additional sensors were not considered a cost-effective addition for this study, given each patient already had a predetermined distance to cover during rehabilitation: the 20-metre corridor.

The system used head movement to target objects in the environment because patients were using their hands to hold crutches. The user was presented with new upgrades, becoming incrementally more powerful and unique to encourage progression.

The scenario escalated to an alien invasion of the city and ended with the patient combatting a giant monster by means of a small puzzle (Figure 1c). The game was designed to coax the participants to continue onward. Each reward they received as they proceeded was more elaborate and exciting than the last. They were provided with multiple layers of visual, haptic, and audio feedback to provide positive reinforcement to continue. The game rewards were decorative items for their avatar that they could earn for use in future play.

#### Procedure

Patients were given a five-minute exposure to the HMD IVR and a similar game to familiarise themselves with the equipment. The patients then used the IVR game during each post-surgery rehabilitation session until their discharge from the hospital, for an average of 10 min per day for 3–4 days. There were slight differences in the number of IVR sessions across the sample because patients differed in the duration of their healing process and, therefore, the length of their hospitalisation. Two physiotherapists received training before the study and worked with the patients each time the IVR was used to ensure comfort with the equipment and avoid possible problems. After each session, patients rated their anxiety and perceived pain. After the clinical trial, interviews were conducted by an experienced interviewer, who was not involved in the administration of the trial. At the end of the trial, another semi-structured interview was conducted with the physiotherapist.

### Outcome assessment

*General Anxiety Disorder-7,* a validated scale for children (8–19 years) to measure self-reported anticipatory anxiety,^38^ was used before each IVR session. Scores for each item ranged from 0 ‘not at all’ to 3 ‘nearly every day’. Scores of 5, 10, and 15 were the cut-off points for mild, moderate and severe anxiety, respectively.

*Pain visual analogue scale,* a validated scale for children (8–17 years) to measure self-reported pain,^39^ was used at the beginning, during and at the end of each session. Scores were recorded by making a handwritten mark on a 10-centimeter line representing a continuum between “no pain” and “worst pain.”

*Semi-structured interviews* were conducted via Zoom with patients and parents followed by individual interviews with the two physiotherapists at the end of the trial. Three closed 10-point Likert questions provided a quantitative measure of patients’ difficulty of use (0 = much too easy, 10 = impossible), pain levels (0 = not at all painful, 10 = extremely painful) and enjoyment of the IVR system (0 = not at all, 10 = I love it). Open-ended questions provided qualitative data on attitudes towards the IVR system and its future deployment, such as difficulty, engagement and enjoyability.

Difficulty was assessed by how easy it was for a patient to set up and learn the IVR system. This included frequency, severity of errors, and difficulty reproducing the therapy and walking. They were asked if instructions were clear, how was it to walk with a walking frame while wearing a VR headset and if the VR game made the physiotherapy easier or harder.

Engagement was evaluated by the extent to which the new intervention was received and aligned with the needs of the target population, in a way that motivated the children. Engagement questions focused on how the system affected their motivation, repeat usage, perceived improvement, and whether they would recommend it to others.

Enjoyment was assessed by how much they were immersed in the game while remaining distracted from anxiety and pain. Patients discussed their enjoyment of the game and whether there were elements that made it more or less enjoyable.

The semi-structured interview with the physiotherapists explored acceptability, usability and perceived walking quality. Staff spoke about the effectiveness of the system in facilitating the desired rehabilitation movements and how it helped with work efficiency. They provided insight into how they felt it affected their patient's anxiety, motivation, and enjoyment during a session.

All interviews were recorded, transcribed and anonymised.

### Analysis

Quantitative data were analysed for descriptive statistics (median [M] and range). The study sample size was small which meant the data did not satisfy a normal distribution, so non-parametric tests were used. The test compared means between repeated pain and anxiety ratings (Wilcoxon signed-rank) along with identification of any sex differences (Mann-Whitney), using the statistical package SPSS v.26.^40^ The hypothesis was that anxiety and pain levels would be greater in session (S) 1 than in S2. Qualitative data were analysed using inductive semantic content analysis^41^ by two qualitative analysts. Analysts conducted analysis of all data separately, then met to discuss and compare themes they had identified in the data and agree on final themes.

## Results

The study included the 15 recruited patients aged 11 to 16 years (*M *= 13.86, *standard deviation [SD] *= 1.29, eight males and seven females) and their corresponding caregivers, including 14 mothers and one father. All participants completed the quantitative measures, and 14 children proceeded to the interview. Participant 14 did not wish to participate in the interview. Table 1 provides details of the participants including the number of IVR rehabilitation sessions, which varied from 2–4 according to individual differences in clinical condition and healing time.

**Table 1.** Patient descriptive data.

Pt ID	Sex	Age	Condition	IVR Sessions
Pt 1	female	13	Meniscus congenital condition.	2
Pt 2	male	14	Distal femur osteochondritis.	2
Pt 3	male	14	Patellar malalignment.	2
Pt 4	female	11	Juvenile arthritis.	2
Pt 5	male	15	Arthroscopy.	4
Pt 6	male	15	Full hip replacement.	4
Pt 7	female	14	Dislocation.	2
Pt 8	female	13	Knee MPFL reconstruction and chondroplasty.	3
Pt 9	male	15	Dislocation.	3
Pt 10	male	12	Both legs surgery, placement of metal plates.	3
Pt 11	female	14	Dislocation.	3
Pt 12	female	16	Hip fracture multiple surgeries.	3
Pt 13	male	14	Knee discoid lateral meniscus cauterisation.	2
Pt 14	male		*Missing interview	4
Pt 15	female	14	MPFL reconstruction	3

IVR: immersive virtual reality; MPFL: medial patellofemoral ligament; Pt: patient.

### Quantitative results: Anxiety, pain, difficulty and enjoyment

Anxiety scores indicated that most of the patients experienced moderate anxiety levels in all rehabilitation sessions (*M *> 10) and close to extreme levels of anxiety (*M *> 15) in the first session (*M *= 14). Significantly lower anticipatory anxiety scores were found in the second IVR session compared to the first (*p *= .041). Although not statistically significant, the anxiety scores reported in the following sessions were also lower than those reported in the first session (Table 2). No significant differences were found in terms of sex.

**Table 2.** Anxiety measures (GAD-7): anticipatory anxiety before the IVR physiotherapy session.

	Anticipatory anxiety before IVR						
	*N*	Minimum	Maximum	*M* (*SD*)	*z*	*df*	*p*
S1	15	7	21	14.40 (4.53)	−2.04	14	.041
S2	15	7	22	11.67 (3.72)			
S3	9	8	20	11.67 (4.06)			
S4	3	7	18	11.00 (6.08)			

GAD-7: General Anxiety Disorder-7; IVR: immersive virtual reality; M; median; S: session of IVR rehabilitation; SD: standard deviation. Answer values: 5 = mild, 10 = moderate, and 15 = severe anxiety.

Pain scores were significantly lower at the end of the second session than at the end of the first (*p *= .011). Although not statistically significant, the pain scores showed a tendency towards higher levels of pain at the beginning of the session (*M*: S1 = 53, S2 = 47, S3 = 48, S4 = 43) compared with pain during the session (*M:* S1 = 45, S2 = 42, S3 = 37, S4 = 40). Pain at the end of the sessions was more variable, as demonstrated in Table 4. Significant sex differences were found in pain scores during the second session (*z = *-1.98, *p = .048*), with females reporting more pain (*M = 54.29, SD = 20.70*) than males (*M = 31.25, SD = *22.16). However, taken as a whole, pain was lower (but not significantly so) in S2 than in the first session (total *M*, S1 = 49; S2 = 42). (Table 3).

**Table 3.** Pain measures (VAS): pain scores before, during and at the end of the IVR rehabilitation.

		Beginning			During			End			Total pain	
	N	*M (SD)*	*z*	*p*	*M (SD)*	*z*	*p*	*M (SD)*	*z*	*p*	*M*	*SD*
S1	15	53 (32.94)	−.98	.325	44.67 (22.40)	−.72	.474	49.13 (30.83)	−2.55	.011	48.94	26.42
S2	15	46.67 (28.20)			42 (23.89)			38.53 (28.59)			42.40	23.74
S3	9	48.33 (29.47)			37.11 (16.09)			48.33 (28.94)			44.60	21.48
S4	3	43.33 (41.63)			40 (39.69)			40 (32.79)			15.50	12.68

IVR: immersive virtual reality; M: median; S: session of IVR rehabilitation; SD: standard deviation; VAS: visual analog scale. VAS scores = 0 to 100%.

**Table 4.** Data extracts: ‘IVR to improve rehabilitation: walking with less pain’ theme.

	ID	Line	Quotation
Patients	2	142–144	Eight [pain rate] at the start just because it were digging into my legs, the controllers, but then when it didn’t dig into my legs, it were like a two.
	8	131–132	On the first session, it was like a seven, but after the second session my pain stayed at around three or four.
	7	225–228	Three (in the pain scale, it's stays that little bit of pain). So then, as I was carrying on, it just seemed to be going away. It was like shooting pain, coming upward from my foot to my knee. […] as I was there and playing on it, there were like little shooting pains coming up. […] When I was playing it, it was like going away.
	6	236	There was no pain whilst I was using the VR.
Parents	3	152–155	He didn't complain of any pain while he was doing it. He did afterwards, but like I can say, it was the first time that he stood up, so he’d only been out of the theatre like 12 h. So I think it was expected to have a little bit of pain. But yeah, it was fine.
	10	296–301	In the first session, I could see he was in quite a lot of pain, but I think In the second session and the third session, I couldn’t believe how fast he was walking, to be quite honest, and I could see he wasn’t apprehensive, wasn’t thinking about the pain and didn’t seem in so much pain as he did the first one, so it was really, it was really nice to see that he had progressed so quickly because he had got something to take his mind off it.
PTs	2	19–21	Before they start the VR session, sometimes the pain can fluctuate, but when they complete the VR, they don’t seem to mention pain or anything along the way.

IVR: immersive virtual reality; PT: physiotherapist.

Quantitative results from the Likert questions showed that patients reported few difficulties (*M *= 3.39, *SD *= 1.80) and perceived little pain (*M *= 3.64, *SD *= 2.20) when using the VR game, with high levels of enjoyment (*M *= 8.82, *SD *= 1.03). Perceived pain differed significantly by sex (*z *= -2.06, *p *= 0.039), with females reporting higher pain scores (*M *= 4.57, *SD *= 2.13) than males (*M *= 2.71, *SD *= 1.99).

### Qualitative results: Participant experience and perceived impact of IVR

**Figure 2.**
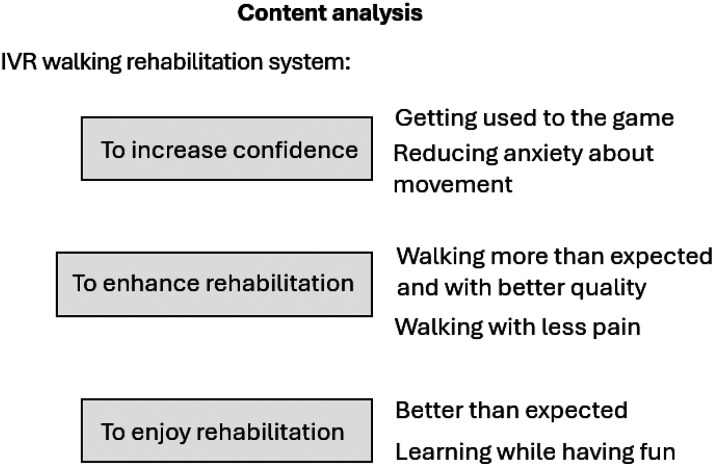
Content analysis: qualitative themes. IVR: immersive virtual reality.

In combination, the content analysis of the information provided by thirty participants (n = 14 patients, n = 14 parents, and n = 2 physiotherapists) generated three main themes plus subthemes: 1. IVR to increase confidence: getting used to the game and reducing anxiety about movement; 2. IVR to enhance rehabilitation: walking more than expected, with better quality and less pain; 3. IVR to enjoy rehabilitation: better than expected and learning while having fun (Figure 2; additional data extracts can be found in the Supplementary Material A). It should be noted that one of the participants (patient 14) declined to participate in the interview for personal reasons.

#### IVR to increase confidence

IVR reduced the anxiety and apprehension experienced by patients when anticipating the pain of movement, which increased their confidence in movement. IVR created some initial anxieties for patients, which were quickly overcome as they gained confidence in the game. Linking IVR use with feelings of anxiety is reflected in the sub-themes*, getting used to the game* and *reducing anxiety about movement*.

##### Getting used to the game

As a novel experience for many (n = 8), some patients expressed feeling a little anxious to start as they didn’t know what to do. This occurred before the system was explained or due to concerns about colliding with furniture. These worries were quickly resolved through explanations, the reassurance of having a physiotherapist beside them throughout and simply getting to experience IVR.

*I did feel a bit nervous. […] When I put it [IVR headset] on and they explained what I was doing*. Patient 8

*I was a little bit anxious as I wasn’t sure exactly what I was doing, but then I felt fine once I got it on.* Patient 11

*It was a bit hard the first time, but it was alright at the end. […] The hardest part for me, or that almost put me off, is thinking I might walk into something, but I weren’t very much concerned in that because I had physio[therapist]s by the side of me and tracking me.* Patient 13

Similarly, one physiotherapist had initial worries about the potential for nausea in patients, but this did not occur.

[…] A *lot of patients after surgery feel quite nauseous and partly down to the anaesthetic they still have in their system after surgery. And we often find, we get patients up and they feel sick, or they vomit, or they collapse, so for the first kind of a couple of VR sessions, I was getting them to stand up and walk before without the headset, and I felt that the VR headset might kind of cause them to feel more nauseous after surgery. But that was not the case.* Physiotherapist 1

*Reducing anxiety about movement*. Parents and physiotherapists talked about the children's reluctance to engage in the exercises because of the fear of pain with which the children often approached rehabilitation exercises, resulting in lower motivation.

With the IVR game, patients were less apprehensive because they had something engaging to focus on. This reduced anxiety and increased confidence for patients to move more quickly:

*It [IVR] didn’t make me worry about my leg. It's calmed down my worrying and anxiety.* Patient 9

*It was easier than just getting straight into it because you’re not focusing that much on it.* Patient 15

Compared with previous experiences without VR, parents (*n *= 3) indicated that their children were looking forward to rehabilitation this time and that the preparation beforehand was therefore much easier:

*He [the child] was very nervous about, […] standing up, putting weight on the leg, very anxious. So, when the VR idea was brought up with him, he was happy. He was excited to start it. […] He was focusing on the game, he had the physios on each side, so he had support with him. And when he took the game off, he was like, ‘Mum, I am in pain, but I’m not like, I’m not nervous, I’m not anxious, and I can’t believe that I’ve walked that much.’* Parent 9

*We’ve had operations before, so this is his eighteenth operation, and this is the first physio session that his anxiety has not been as bad […] I spend a lot of my time kind of trying to prepare him to go to physio, and he's not looking forward to it, but this time because [physiotherapist name] had kind of told him about this, he was actually looking forward to it, so for me as a parent it was easier because it was giving him something to focus on […] and he's moved a lot quicker than he would have generally moved before. […] whereas it usually would have taken a lot more sessions to do that because he's overthinking things. He's anticipating the pain, whereas this time, he's not had that to think of because he has been doing something fun and had something else to concentrate on.* Parent 10

Physiotherapists commented on the value of the IVR tool in reducing anxiety and pain levels, allowing the patients to focus on something else while still managing to produce the necessary movements.

*Patients after surgery are very kind of anxious and worried, and they are in pain, and they know that when we do mobilising and do physio sessions, it will increase their pain. But by using the VR and playing a game and distracting them, it's of great benefit in increasing their confidence and getting them to do what we want them to do and that is to aid their recovery.* Physiotherapist 1

The physiotherapists noted that frequent exposure to surgery and rehabilitation in the past tended to increase their patients’ anxiety levels, and they were particularly pleased with the anxiety-reducing impact of VR in this group.

*One patient who had numerous surgeries in the past […] He was in quite a lot of pain, and he was very nervous about walking, […] once he put the headset on he managed to walk really well, and completely distracted him and gave him that confidence that he needed. After he did his first and second session […] he’d made so much progress in that small amount of time with the VR than he did without it.* Physiotherapist 2

#### IVR to enhance rehabilitation

Patients (*n *= 11) reported that rehabilitation with IVR was easier than without. Eight out of 14 indicated that they had managed to move better with the IVR system than they had expected because they were distracted from the difficulty of movement. Patients spoke of walking further distances, walking more quickly, making better quality movements, and walking with less pain.

*Walking more than expected with better quality:* Four out of 14 patients mentioned walking a greater distance than in pre-IVR rehabilitation sessions. One indicated doing it in less time (Patient 1), and another with little effort (Patient 5). They also perceived improvements in their walking, with more natural movement (Patient 10), walking on the whole foot or weight-bearing more evenly (Patient 2). The improved movement patients perceived may have resulted from being less fearful and more engaged, immersed in and distracted by the game:

*I were [walking] like on my tiptoes. But when I were doing it with VR, I could put my whole foot down and not be scared.* Patient 2

*When I took it off, I was surprised at how far I’d walked. [Without it] I’d struggle to walk and probably be at the hospital for a couple more days.* Patient 13

Parents and physiotherapist 1 also saw improved movements*:*

*He had an operation on the other leg […] and the VR wasn’t involved then, so this time, he seemed to get up a lot quicker and get on with it [rehabilitation] and do a lot more as well than he did compared to last year.* Parent 3

*I think with the VR headset because it distracts them […] the patient then just walks normally and he's not kind of worried or thinking about their operated leg. So by getting them to do all that and to walk normally it's a massive progress for them.* Physiotherapist 1

*Walking with less pain*: All parents (n = 14) considered that their children were not in much pain. Parents and children described a pattern of how they experienced pain: more pain in the initial than later IVR sessions, reduced pain during play, and a resumption of pain after play. They described being aware of pain returning, particularly after the first session, but this pain was no more than regular post-rehabilitation pain. However, several children described pain at the location where the controllers were attached to their legs.

Children, parents and physiotherapists were surprised and pleased by the reduction in pain and the resulting improvement in movement due to the IVR experience (Table 4).

#### IVR to enjoy rehabilitation

Participants were very positive about the IVR experience and impressed with its impact on rehabilitation. Most patients (*n *= 13) expressed high satisfaction and would recommend IVR to others undergoing physiotherapy. Eight out of 14 reported that the rehabilitation would have been worse without the IVR. Specifically, it would have been less enjoyable (Patients 2, 4, and 8), less effective (Patient 2), more fear-inducing (Patient 6), stressful (Patient 12), painful and difficult (Patient 7).

Most of the patients (n = 8) indicated that they enjoyed the IVR rehabilitation game more over time, such as at the end of the session, because they liked the gameplay: “when I was killing the boss, and it was exciting to see what score I got” (Patient 2) or “because it was more comfortable” (Patient 3). They enjoyed the second and third sessions more (Patients 4, 11, 12, and 15), or they enjoyed last two sessions most because they knew better what to do (Patients 8 and 10). Two indicated that they enjoyed all sessions the same amount (Patients 1 and 13).

Four out of 14 reported enjoying the first session more because they did not know what to expect (Patients 5, 6, and 9) or were surprised by the distance they had walked for the first time (Patient 7). One patient reported feeling it was a bit more boring towards the end of the therapy because he had done the level already (Patient 5).

*Better than expected*: For most participants (n = 9), the experience of IVR exceeded their expectations in terms of enjoyment:

*I just thought it’d be like a hospital game. Like really boring. Nothing to do. The VR itself was good, an interesting game.* Patient 5

Children (*n *= 13) reported that the game helped them with motivation and commitment to rehabilitation:

*It motivated me to do more than what I hoped I could do. And it pushed me to do better.* Patient 6

*It affected me in a good way with motivation because I walked around and realised that I can still do stuff with my leg without the VR. I can do, like do more, and I think VR proved to me. Made me more confident.* Patient 9

One physiotherapist also saw increased motivation:

*I think it increases their confidence because, when we go in the morning for the first session, they want to participate in the second session willingly, all ready to go.[…] And it [IVR] gives them a goal and purpose. Just for them to engage in something that is just not walking, it is more walking with an aim.* Physiotherapist 2

*Learning while having fun*: The children were clear that their enjoyment of the game supported the learning process and helped them progress. Patients (n = 8) indicated that having a game they found exciting and fun helped them greatly in their rehabilitation, to want to continue and keep going until they recovered. Parents (n = 8) said that they observed their child enjoying IVR and having fun. One PT supported that IVR made the physiotherapy more enjoyable for the patients and their parents:

*For a parent to witness or observe their child in pain, it is very difficult. But when you see your child playing a game and being interactive with this VR headset and having fun and not in pain, that's very positive for a parent to experience […].* Physiotherapist 1

## Discussion

Anxiety outcomes demonstrated moderate to extreme levels of anxiety in the patients before post-surgery rehabilitation sessions. This was largely due to the fear of pain, reducing children's confidence in their rehabilitation exercises and their ability to regain movement.^13,14^ IVR significantly reduced anticipatory anxiety levels between the first and second sessions, showing that IVR use in S1 had a profound impact on the anxiety with which they approached S2. Qualitative findings suggested a particularly positive shift in pre-session anxiety for those children who had already undergone several operations and for whom anxiety had previously been very problematic. It is recommended to consider this result in future research, to inform a larger study and to identify participants who can benefit most from this new system.

Previous studies have used IVR to manage anticipatory anxiety preoperatively to familiarise patients with the operating room environment and procedures^17^ or focused on relaxation and helping patients concentrate on rehabilitation tasks.^35^ The current study utilised the IVR game to distract, directing attention away from a stressor and towards a positive focus, as done in previous studies.^18,42^

Some anxieties were reported before using IVR for the first time, so it would be beneficial to offer an opportunity to familiarise the patient with VR technology.^12^ This could be in the form of an initial short video showing what to expect from VR. A simple VR demonstration could follow, in which the user does not need to strap the HMD to their head but instead hold it in front of their eyes so they can quickly disengage to maintain control over the situation.

IVR has been shown to reduce the pain of rehabilitation by shifting attention away from incoming painful sensory signals and maximised recovery by increasing motivation and adherence.^16,19,23–26,43^ In this study, patients experienced a significant reduction in pain using IVR. Evidence supports the use of IVR with HMD in achieving total immersion, reducing pain and enhancing outcomes, compared with non-immersive interventions which are limited in terms of interaction and sensorimotor contingencies.^44,45^ However, patients indicated that pain resumed at the end of IVR rehabilitation sessions. This was generally reported as a level comparable to their previous post-rehabilitation pain, indicating that usual analgesic measures would be effective in controlling it.^46^

Although no major sex differences were found, females reported higher perceived pain than males during gameplay. It may be that females were somewhat less engaged than males by the content of the game; however, qualitative comments suggested that this was not the case. There is some evidence that the sexes differ in their sensitivity to and risk of clinical pain.^47^ The system used for the current study offered customisation in the design of the patients’ virtual legs, giving them the freedom to choose how they looked according to their individual preferences. The team will continue to consider pain differences by sex when using IVR.

Parents frequently reported the previous stress they had experienced resulting from their children's anxiety and pain. Indeed, evidence suggests that negative emotions can be transmitted during hospitalisation between patients and their caregivers and that this can affect recovery.^48,49^ In contrast, IVR proved helpful in relieving their children's anxiety, pain and stress, offering instead an enjoyable experience they could share with their children. Given the emotional bond between child and parent, future studies conducted by the team will include measures of parental anxiety.

IVR proved suitable for a wide range of patients and successfully replicated usual rehabilitation exercises of standing, weight-bearing on the affected limb and walking 20 metres. In line with previous adult-based studies, participants in the current study indicated that IVR improved lower limb function after surgery, facilitating movement often more quickly and effectively when compared with previous post-surgery rehabilitation without VR.^21,27,29–31,33–35^ The minimisation of perceived task difficulty is an important finding. If patients are more willing to comply with early rehabilitation, this can improve the physical outcomes of rehabilitation,^12,13,24^ facilitate earlier discharge and avoid long-term conditions, resulting in cost savings for families and the NHS.^7,9^

Future iterations of the game will increase the number of scenarios and introduce new skills, keeping the approach of maintaining a positive impact over time and engaging patients to commit to treatment.

The system setup did not present difficulties for participants, and no system glitches were reported. However, patients reported discomfort and pain resulting from the controller attached to their affected leg. This will be addressed in future trials, and a more comfortable attachment holster will be used.

Finally, it should be noted that although positive results were observed for this intervention and overwhelmingly positive comments were received from all three sets of participants, this was a proof-of-concept study, employing a limited sample. A notable limitation of the study is that no pre-surgical functional measures of lower limb performance were taken, as recruitment took place after surgery and the patients were starting from no lower limb mobility. There was also no control group due to the exploratory nature of this preliminary study. A future clinical trial to establish effectiveness will include a large sample, a comparison group and control variables. This will allow for more robust data analysis and greater generalisability of the findings. Although VR safety precautions were adopted in the current study, these would be formally recorded in follow-up clinical trials.

## Conclusion

This IVR system is the first of its class for paediatric lower limb postoperative rehabilitation. This small-scale study contributes to the emerging body of research on the use of medical technology for children with physical motor impairments. It presents the potential of IVR for this patient group whilst highlighting the challenges faced in the deployment of IVR for lower limb rehabilitation.

The co-designed IVR system developed in this study aimed to reduce anxiety and pain during paediatric lower limb postoperative rehabilitation. The outcomes of this proof-of-concept study helped to establish that the IVR system assisted in reducing anxiety and pain along with promoting weight-bearing movement after lower limb surgery in young people. These preliminary results will guide the protocol for subsequent clinical trials to improve the system for use in a future large-scale multi-site study. These studies will aim to verify the system's efficacy, safety and effectiveness, both in the clinical setting and with the potential as a home-based lower-limb rehabilitation solution.

## Supplemental Material

sj-docx-1-prm-10.1177_18758894241313093 - Supplemental material for Immersive virtual reality rehabilitation after lower limb surgery in paediatric patients Supplemental material, sj-docx-1-prm-10.1177_18758894241313093 for Immersive virtual reality rehabilitation after lower limb surgery in paediatric patients by Ivan Phelan, Alicia Carrion-Plaza, Penny Jayne Furness, Jack Parker, Nicolas Nicolaou and Paul Dimitri in Journal of Pediatric Rehabilitation Medicine
