# Supplementary material for: Immersive virtual reality rehabilitation after lower limb surgery in paediatric patients
Source: J Pediatr Rehabil Med. 2025 Jan 31;18(1):30–41. doi: 10.1177/18758894241313093 (PMC13292700; doi:10.1177/18758894241313093)
Supplement: sj-docx-1-prm-10.1177_18758894241313093 - Supplemental material for Immersive virtual reality rehabilitation after lower limb surgery in paediatric patients [file sj-docx-1-prm-10.1177_18758894241313093.docx]

Supplementary material A. Participant testimonies.

1. Testimonies about IVR to increase confidence: reducing anxiety about movement.

| **ID** | **Line** | **Quotation** |
| --- | --- | --- |
| Pt 8 | 149  151  158 | I did feel a bit nervous.  Like everything.  When I put it on and they explained what I was doing, I felt less nervous. |
| Pt 9 | 190-191  134-135 | First I was nervous, but when they told me what we were going to do, I think I got more excited. And it’s because wanted to play different games. (…)  It made me less anxious, you know, it didn’t make me worry about my leg, and all that. It just, it’s calmed down my worrying and everything and anxiety. |
| Pt 5 | 184-190 | I was a bit anxious. Just about everything. INT: When you started playing the game, your anxiety of that feeling that you have it increased or decreased? Pt: It definitely decreased. |
| Pt 6 | 149  259-260  270 | I felt fine.  INT: Have the VR help you to get started more easily than for example without the VR when you did your exercise?  Pt: yes, because it helped me to interact more and encourage myself to do it. |
| Pt 7 | 254-257 | I was a bit anxious at the start, but then, I was all right. INT: When you first started using the game were you nervous but then it went away? Pt: Oh, yeah, a bit, and weren’t anymore. |
| Pt 11 | 267-268 | I was a little bit anxious as I wasn’t really sure exactly what I was doing but then I felt fine once I got it on. INT: Do you think the VR helped you get started more easily? Pt: Yes, definitely. INT: And when you started playing the game did the anxiety decrease or increase? Pt: Yes, it went. |
| Pt 12 | 263  269-281 | I was a bit anxious, I didn’t know what it was going to be like at all.  INT: Was the VR helped you to get started more easily?  Yes, yes. INT: How the VR helped you if you can think about that? Pt: Well I wasn’t really concentrating on the pain or how hard it might be to walk or anything, just concentrating on the game. INT: When you started playing the game did your anxiety decrease? Pt: Yes, yes, it went away. |
| Pt 13 | 251-255 | It was a bit hard the first time but it was alright at the end. INT: So did you feel a bit nervous about what will be in the VR? Pt: Yes, I was thinking that but when I was on it was okay.  […] The hardest part for me or that almost put me off is thinking I might walk into something, but I weren’t very much concerned in that because I had physio’s by the side of me and tracking me, but everything but that it was perfect for me. |
| Pt 2 | 111-113 | I were trying to walk with it, I were like on my tiptoes. But when I were doing it with VR, I could put my whole foot down and not be scared. |
| Pt 10 | 281-283 | The first time I was excited, but a little bit nervous because I weren’t quite sure what I was meant to be doing, but as time went on through the game I got a lot more confident. |
| Pt 2 | 50-51 | You feel more confident walking on your leg, you get used to it more. |
| Pt 15 | 60-61 | I think it was easier than just getting straight into it because you’re not like focusing that much on it. |
| Pr 9 | 77-101 | I found it amazing, because this was a boy who was very nervous about, because obviously he’s had surgery, so he was very nervous about you know, standing up, putting weight on the leg, very anxious. So, when the VR idea was brought up with him, he was happy, actually, he was excited to start it. He didn’t even realise when he started the game that he was, he actually walked, I think did they say 15, 15 kilometre. He was focusing on the game, he had the physios on each side, so he had support with him. And when he took the game off, he was like mum, I am in pain but I’m not like, I’m not nervous, I’m not anxious and I can’t believe that I’ve walked that much. I said, yes, you walked it. And he was very happy with it as well, I think the experience with the VR game, you know, he enjoyed it very much. I think he would have liked more sessions on. Because we had to come home, so you know, he had one session on Wednesday morning and then he had another session, he had two sessions together on Thursday, so, Thursday afternoon, he had one where he was walking with his frame and then another session when he was walking with his crutches. So, we managed to do two sessions with him together and he actually walked a good distance and I don’t think he would have done that, if he didn’t have the VR game. And I think he would have been a lot more anxious (…), I don’t think he would have done it. So, the VR game is a very big help and I think improvement wise, I think it’s amazing, it doesn’t need any improvements. I think it will help a lot of children. You know, so yes, I would have loved to have had a try on it, I was looking at the computer screen when he was playing it and I was like, oh wow, that looks really good. |
| Pr 10 | 88-107 | I think for me as a parent we’ve had operations before, so this is his eighteenth operation and this is the first physio session and his anxiety has not been as bad because obviously we’ve had so many, he knows what pain and rehabilitation is going to be like after, so for me as a parent I spend a lot of my time kind of trying to prepare him to go to physio and he’s not looking forward to it, but this time because (physio name) had kind of told him about this, he was actually looking forward to it, so for me as a parent it was easier for me because it was giving him something to focus on that he was going to look forward to rather than not look forward to, so it was brilliant for me because you know usually every session I have got to try and say you know, trying to jeer him up a bit and say you know, come on we can do this, whereas all three sessions he’s looked forward to doing it and he’s moved a lot quicker than he would have generally moved before. I mean in the space I can’t believe how quickly he’s walked in the space of three sessions whereas it usually would have taken a lot more sessions to do that because he’s overthinking things. He’s anticipating the pain, whereas this time he’s not had that to think of because he has been doing something fun and had something else to concentrate on, so I think for me as a parent watching it whereas I have not done it, I have loved it because it’s made it more enjoyable for him and hopefully if the hospital move forward with it next year when he goes through the same operation it will be the same experience looking forward to rehabilitation period because he’s going to have something to look forward to doing. |
| Pr 15 | 172-176 | I think it was good because it distracted her, whereas she was apprehensive about walking and putting her weight on her leg, that sort of distracted her to concentrate and concentrate what was on the virtual thing. Sort of like took her mind off that worrying you know and she was able to do her physio better. That’s what I saw when I was there. |
| PhyTs 1 | 138-143 | With the VR headset it kind of gives them that visual stimulus and something to focus on that they don’t even have, they don’t have the time to worry anymore and think so they just kind of, they’ve got this purpose to focus on what they need to do and then before they know it they’ve finished the game then they’ve realised oh I’ve walked this far, it’s not too bad actually. It works really well. |
| PhyTs 2 | 130-135 | There’s obviously been a few that are, have been anxious to obviously participate with the VR, I think that’s more so like pain than actually getting up. But obviously once they’ve put this head set on and realised they’ve completed a game and you show the distance that they’ve walked it gives them that boost of confidence and then the next time round they are not as anxious and they want to do it. |
| PhyTs 1 | 164-171 | Anxiety and pain are very closely linked so by reducing the anxiety they are then reducing their pain as well so I think it has worked tremendously well. We often notice that when they do the VR they probably, every time they take a step forward and put weight on that operated leg they will be like, they will not complain too much but then as soon as you remove the headset and get them to walk either to the wheelchair or back to their bed then they often say oow, oow, or they then report some sort of discomfort. But whilst they had the headset on they’ve not reported that. |

Pt = patient, Pr = parent, PhyTs = physiotherapist, INT = interviewer

2. Testimonies about IVR to enjoy rehabilitation: Better than expected

| **ID** | **Line** | **Quotation** |
| --- | --- | --- |
| Pt 5 | 24-25  32-33  38-42 | I just thought it’d be like a hospital game. Like really boring. Nothing to do.  The VR itself was good, an interesting game.  This one was really fun, but it was a bit harder because I was only using my legs and not my hands for anything. Compared to the other ones where I used like my whole body so, it’s quite different. |
| Pt 6 | 71 | It was more engaging than I thought it would be. |
| Pt 10 | 47-49 | I think it was better because at first when the physio’s came round to tell me I didn’t like quite think about the feeling of walking and but when I put my headset on I just completely forgot about walking and just try and play the game. |
| Pt 3 | 175-178 | (I recommend it) Yes. Well, because I’ll just tell him that it will help them take their mind off the pain and everything and help them recover faster. |
| Pt 4 | 160-161 | (I recommend it) Yes, because it really helped and it made it easier and enjoyable, more enjoyable to do it. |
| Pt 5 | 225-228 | (I recommend it) Definitely, yes. It’s interesting. It’s like something you should try if you have the chance. It just makes it easier and more fun. |
| Pt 6 | 335 | (I recommend it) Yes. Because it can also motivate them to do more. |
| Pt 7 | 309-310 | (I recommend it) Yeah. Because you don’t realise how far you walk until you actually take the VR off. |
| Pt 8 | 195 | (I recommend it) Yes. Because I think it really helped me. |
| Pt 9 | 232-233 | (I recommend it) Definitely, yes. It would also help with motivation and his confidence, and it probably would entertain him. |
| Pt 10 | 324-326 | (I recommend it) Yes. Because there’s nothing to be scared about and you are not even thinking about anything except from the game and you are just focusing on about seeing what there’s more to come in the game in like progressing. |
| Pt 11 | 321-322 | (I recommend it) Yes. Because I think it would really help people, it’s really helped me and it doesn’t make you feel as anxious and it’s more motivating. |
| Pt 12 | 330-335 | (I recommend it) Yes, I would recommend it. Well I know some of my friends would panic about doing physio exercise so it would definitely take their mind off of it. |
| Pt 13 | 298-299 | (I recommend it) Yes. It would just help them start walking and be like you wouldn’t really feel the pain because you’re more into the VR. |
| Pt 15 | 387-388 | (I recommend it) Yes. Because I think it would help them to get their mind off it and just get straight into it rather than them just being nervous about it. |
| Pt 2 | 205-207 | I wouldn’t enjoy it as much and I probably wouldn’t have learnt from it. So I’m learning more how to walk on it because I’m walking further than I thought I did. |
| Pt 4 | 149-151 | I think it would be a little bit less enjoyable, because your mind would only be focused on the exercise, so I think it would be, it's better with the VR. |
| Pt 6 | 326 | I think without the VR I would be more worried of what I could do. |
| Pt 7 | 303-304 | I think it would have been painful, like I wouldn’t have been able to do it. |
| Pt 9 | 178-180  184-186 | If I didn’t have it, I think, yes (more painful), I don’t think I’d have got on. I don’t think I would have walked, I probably would have kept worry and more anxious and pain. Because, I think I’d be sitting all day and not doing anything. Whereas the VR, I’d be moving around, making my leg more active. |

Pt = patient, Pr = parent, PhyTs = physiotherapist

**3. Testimonies about IVR to enhance rehabilitation: walking more and better.**

| **ID** | **Line** | **Quotation** |
| --- | --- | --- |
| Pt 5 | 55-57  60-61 | I’ve done physio before and it was less professional. Can I say it was less intense, it was just like you’re out there playing a game and actually getting work done.  I think the effort was about the same but I’ve **got more walking done**. |
| Pt 6 | 194  198-199 | It helped me to do more than what I thought I could be able to do.  Because like it pushed me to be able to like because it stops me thinking about my leg and it **gets me more involved**, and it helps me. |
| Pt 7 | 138-139 | I didn’t realise how I walked in that far until I took it off. |
| Pt 12 | 99 | I didn’t realise I was **walking as much as I was.** |
| Pt 1 | 93 | I think it helped because it made it **go faster.** |
| Pt 10 | 37-39 | It’s a way of **getting better movement** in you without thinking about the pain and where you are walking and it’s just and you are just concentrating on something different than your feet. |
| Pt 4 | 89-90 | It made it **easier to walk** and made it easier because my mind was occupied and think about it as exercise as much. |
| Pt 9 | 26-27 | Didn’t have to worry about my leg in pain and all that. I was focusing on one thing. |
| Pt 8 | 111-112 | Easier. Because I’m not focussing too much on what I’m doing, but it like keeps my mind occupied while I’m trying to walk. |
| Pt 15 | 251  255-256 | I think it made it easier.  Helps you just like distracted from, wouldn’t be as scared to do it, you just do it because you’re too busy concentrating on the game. |
| Pt 3 | 96-97 | It just it took my mind off the pain instead of just walking in the corridor without anything really. |
| Pt 7 | 197 | It helped me put weight on my knee when I was walking. |
| Pr 3 | 60-63 | Well, we can compare this because (patient name) had an operation on the other leg last December. And the VR wasn’t involved then so this time doing his physio he seemed to get up a lot quicker and get on with it and do a lot more as well than he did compared to last year. |
| Pr 5 | 84-87 | He just had surgery two days ago so the fact that he could be distracted while he was doing something that was quite painful for him, that was really helpful and I do think he went a lot further. I think if he wasn’t doing the VR, I don’t think he would’ve gone that far. What we see would’ve felt less motivated to do them. |
| Pr 7 | 146-160 | (Patient’s name) suffers in a lot of ways, she has ADHD (difficulty sustaining attention), so her attention span is very, very short. And, she doesn’t, if she thinks that she’s not going to enjoy something, then she won’t do it. So, to do the VR, and see how actually she was, what’s the word I’m looking for, distracted.  I mean, thrilled in it, she actually did a lot more than what I expected her to do.  So, especially the first session. I think the only reason why she stopped, was the pain that she was getting.  But the distraction of the game allowed her to get to the point where she got with walking, without realising she’d gone that far. |
| PhyT 1 | 38-41 | INT: Did the game get the patients to do the movement they needed for their rehabilitation? PhyTs: Yes, so the kind of, one of the key markers we want to improve is their walking with generally with a walking aid. So, by having the sensors on the knees and getting them to do what they need to do that kind of coincided with that and that worked really well. |
| PhyTs 2 | 43-44 | They need to be able to walk a certain distance before they are obviously happy for physio discharge, so this encourages them to walk a fair distance. |

Pt = patient, Pr = parent, PhyTs = physiotherapist, INT = interviewer

**4. Testimonies about IVR to enhance rehabilitation: walking with less pain**

| **ID** | **Line** | **Quotation** |
| --- | --- | --- |
| Pt 2 | 142-144 | Eight at the start just because it were digging into my legs, the controllers, but then when it didn’t dig into my legs, it were like a two. |
| Pt 8 | 131-132  134 | Well, on the first session it was like a seven, but after the second session my pain stayed at around three or four.  It’s like an irritable pain. |
| Pt 7 | 227-228  238-243 | Three, It’s stays, that little bit of pain. So then, as I was carrying on, it just seemed to be going away.  It was like shooting pain, coming, upward from my foot to my knee. So, like, as I was there and playing on it, there were like little shooting pains coming up. INT: When you started playing the VR game, did the pain start to decrease or was it the same? Pt: When I was playing it, it was like going away. |
| Pt 11 | 205-211  232-234 | I wasn’t really thinking about the pain as much so it was quite a lot less than what it was when I didn’t have it on. I’ve got this, there’s this experience of like the beginning, at the beginning it was like 80% pain, then it went down to, this was the first session. So in the middle it was around 50% and then it, at the end as I’ve been walking for the first time it went back to up to 90%, but in the middle it went down.  It was just my knee that was painful, nothing, like none of the things that they strap round, I know the physio said people find that discomfort. I found that fine, it was just my knee that was. |
| Pt 6 | 245 | There was no pain whilst I was using the VR. |
| Pt 12 | 236 | I didn’t really feel uncomfortable, I wasn’t in any pain. |
| Pr 1 | 154-161  169-173 | She didn’t look much in pain to me even though it was, and walking after the operation. INT: Did you see the pain that she’s describe? Pr: Yes, because like at the beginning because she was concentrating to stand up and making the right movement at the beginning. But as she started, she was more focusing and I was just watching her face impression. She was just more focused on killing the aliens.  I just think that because of the distraction, so they don’t focus on the pain, it definitely helps. And especially because it was enjoyable, they wanted to do it further and more and go to the end, so they wouldn’t think about the pain. |
| Pr 3 | 152-155 | He didn't complain of any pain while he were doing it. He did afterwards but like I can say it was the first time that it stood up so he’d only been out of the theatre like 12 hours. So I think it was expected to have a little bit of pain. But yeah, it was fine. |
| Pr 4 | 142-143 | She didn't feel any pain. She didn't complain even once so yeah. |
| Pr 6 | 298-299 | He told me he didn’t have any pain. Yes, I think the VR helped with not having any pain because it takes the mind off the actual injury, so it helped him a lot more. INT: He used to experience pain when he do rehabilitation without the VR?. MUM: Yes. |
| Pr 7 | 278-288 | So, I thought she didn’t have as much pain during the VR session, to what she did when she had normal physio sessions. I found that the VR was a very good distraction for her. So, it allowed her to not think about the pain, but doing a physio at the same time. During the VR sessions, she didn’t seem to be in any pain, for quite a while. And then the pain started kicking in. While with without the VR, the pain was there throughout. |
| Pr 9 | 206-221 | I think the pain was there, but it took his mind off, because he was more focused about killing the monsters. But yes, he had pain and then when we got back from the session, he requested some pain killers. But to be fair, we knew, because he’d walked quite a lot of distance, we knew that there would be pain. But actually, he wasn’t in as much pain as I thought he would be, I thought he would be in quite a lot of pain with his knew, because of the distance that he walked. But he was actually talking to me more about the VR game than the pain. so, I think the VR game took his mind off it. and he was actually quite proud when we showed him how long he walked and he thought, he said, wow, did I walk all that, we said yes, you walked all that without knowing. But yes, he is on some strong pain killers, he’s on morphine and tramadol, paracetamol and ibuprofen. Which he did have after the sessions. But he did not complain about the pain, he was more focused on the VR game and what, you know, what he played and what he did. so I think it’s a great asset to have this there for them, for children that are you know, a bit quiet, you know that are a bit nervous, a bit anxious, because it takes the mind off things, so yes, I think it was amazing. |
| Pr10 | 296-301 | In the first session I could see he was in quite a lot of pain, but I think throughout the second session and the third session I couldn’t believe how fast he was walking to be quite honest, and I could see he wasn’t apprehensive, wasn’t thinking about the pain and didn’t seem in so much pain as he did the first one, so it was really, it was really nice to see that he had progressed so quickly because he had got something to take his mind off it. |
| Pr 11 | 286-288 | I didn’t feel that she had a lot of pain during the session at all. I know after when she got back on the bed after she said it was painful, but during it, it was absolutely fine. |
| Pr 15 | 348-354 | Yes, I didn’t think it was so much pain. She had apprehension about moving because she didn’t know how she would hurt. It was the first time, but the game was a distraction, and I don’t think she was in pain. After a while, she started to feel dizzy, stood up and walked, but I think that probably the game, well definitely the game helped her do it for longer. Because she was concentrating on that as opposed to thinking “oh, I feel dizzy” or “my knee is hurting”. I think it helped her to focus. |
| PhyTs 2 | 19-27 | Before they start the VR session, sometimes the pain can fluctuate, but when they complete the VR, they don’t seem to mention pain or anything along the way. They don’t need to rest, they are using the VR, and I think it increases their confidence because, for example, when we go in the morning for the first session, they want to participate in the second session willingly, all ready to go. So I think it just increases their confidence as well. And it gives them a goal and purpose. Just for them to engage in something that is just not walking, it is more walking with an aim. |

Pt = patient, Pr = parent, PhyTs = physiotherapist, INT = interviewer

**5. Testimonies about IVR to enjoy rehabilitation: learning while having fun.**

| **ID** | | **Line** | **Quotation** | |
| --- | --- | --- | --- | --- |
| Pt 2 | | 33-34  48-50 | Fun, it’s another funnier way to learn how to use crutches or a frame.  If it weren’t a VR game and you will just walk, it’d be kind of boring, but since there’s actually so much to do, to get to the end to try and kill the boss. | |
| Pt 3 | 19-20 | | It was like it made it more fun to learn how to walk again with crutches and everything else. |  |
| Pt 5 | 54-55 | | I think just the game itself made it more enjoyable, it’s not like I’ve done physio before. |  |
| Pt 10 | 199-201 | | It helped me about like having fun. It helps you in different ways to progress because you are having fun and not worrying about anything. |  |
| Pt 12 | 55-56 | | It was a fun game to play while I was doing my exercise. |  |
| Pt 5 | 18-19 | | It’s interesting, but when you’ve done it a few times, it becomes a bit boring. |  |
| Pt 7 | 89-90 | | Because sometimes it kept on like glitching out, so then I got really frustrated. |  |
| Pr 11 | 306-307 | | I think it’s enjoyable and fun, so it pushes you to want to do it more and get up because it’s fun to do. |  |
| Pr 6 | 158-160 | | He said he liked kicking the aliens and pushed himself to do a bit more because it was more fun than normal physiotherapies, like it can get boring. |  |
| Pr 10 | 131-137 | | So watching him do it made, you know I was smiling the whole time because I knew he was doing something that he really enjoyed and he wasn’t going to be focusing on his pain that he was going to be having although his first session it was really, painful, I could see that he was pushing through it because he was enjoying it. |  |
| Pr 13 | 105-106 | | It was really good. It took his mind off his leg, his injury, the pain, so it was a good diversion and a good diversion for me as well, so yes, it’s good. No, it’s just everything, you know it works, whatever, you know how to get kids to walk again, and if they’re in pain, it will take their mind off it, so no, that’s it. |  |
| PhyTs 2 | 106-108  200-201 | | I just think we’d be doing the mobilisation anyway but adding the VR just makes it a bit more fun for them to want to walk rather than focus on pain.  (…) they all seem to enjoy it, everyone that we’ve done. |  |
| PhyTs 1 | 196-198 | | Playing the game and getting their interaction with it, it’s making it more fun because, like (staff 2 names) said, they normally have to do this walking anyway, but because it’s made more enjoyable, it’s definitely having a positive effect. |  |

Pt = patient, Pr = parent, PhyTs = physiotherapist
